# Supplementary material for: Seminoma and Embryonal Carcinoma Footprints Identified by Analysis of Integrated Genome-Wide Epigenetic and Expression Profiles of Germ Cell Cancer Cell Lines
Source: PLoS One. 2014 Jun 2;9(6):e98330. doi: 10.1371/journal.pone.0098330 (PMC4041891; doi:10.1371/journal.pone.0098330)
Supplement: File S1 — ZIP file containing DMRforPairs output for significant regions. Please start from the html files. (ZIP) [file pone.0098330.s008.zip › figures/42048.pdf]

RegionID: 42048, chrX:134186198−134186640−M_values

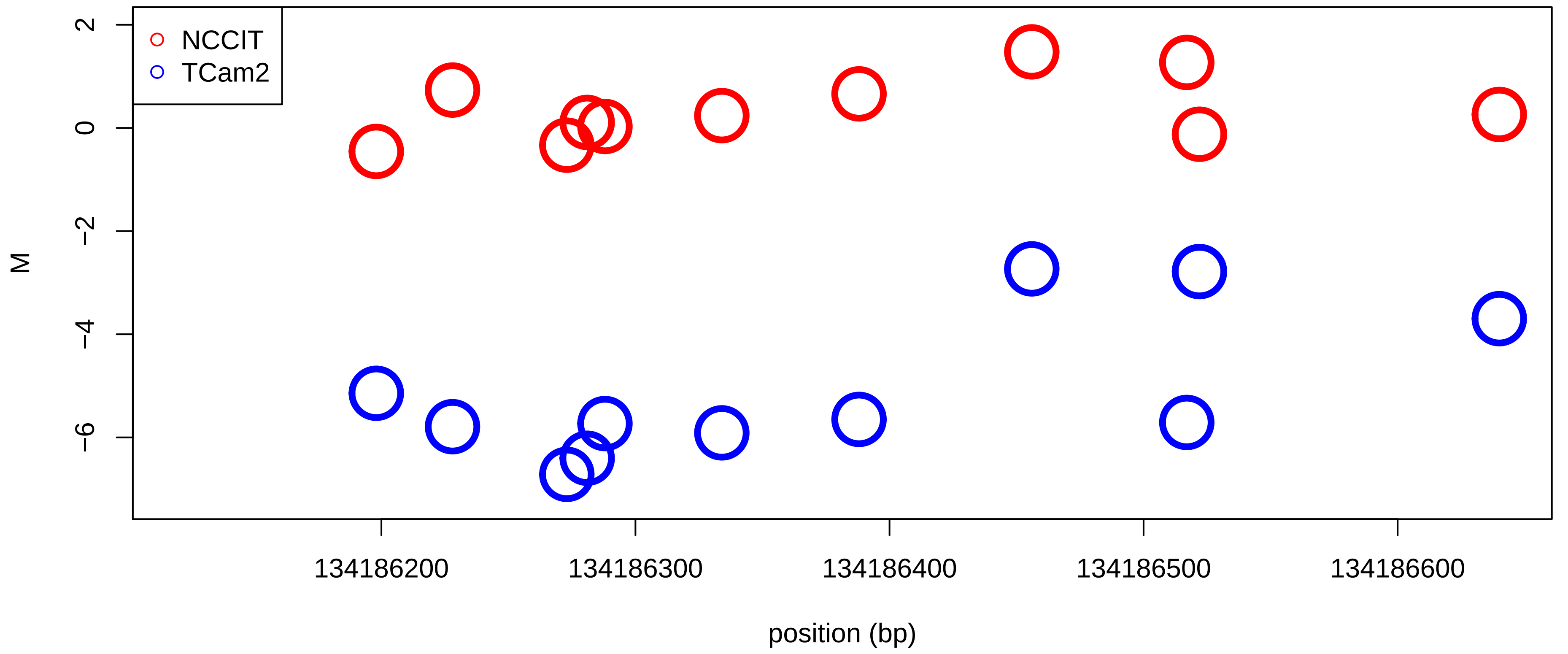

RegionID: 42048, chrX:134186198−134186640−Beta_values

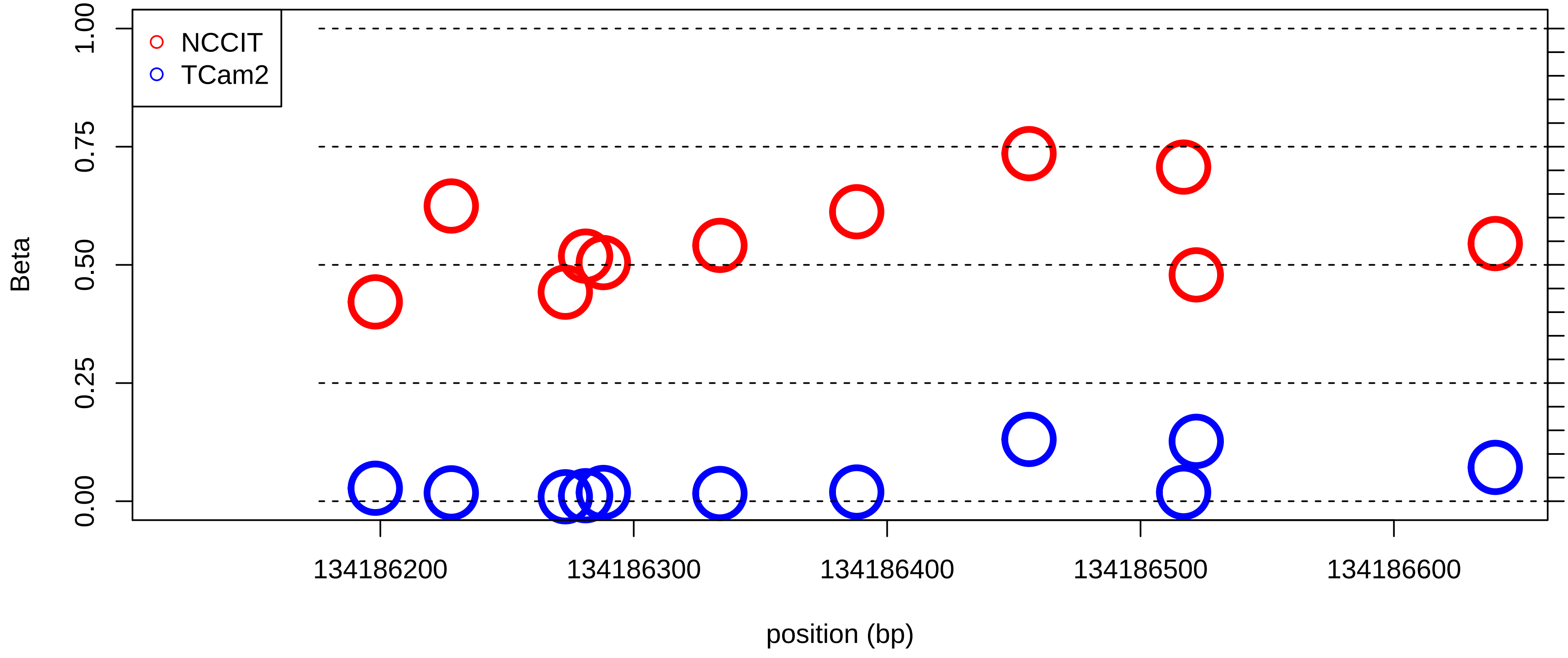

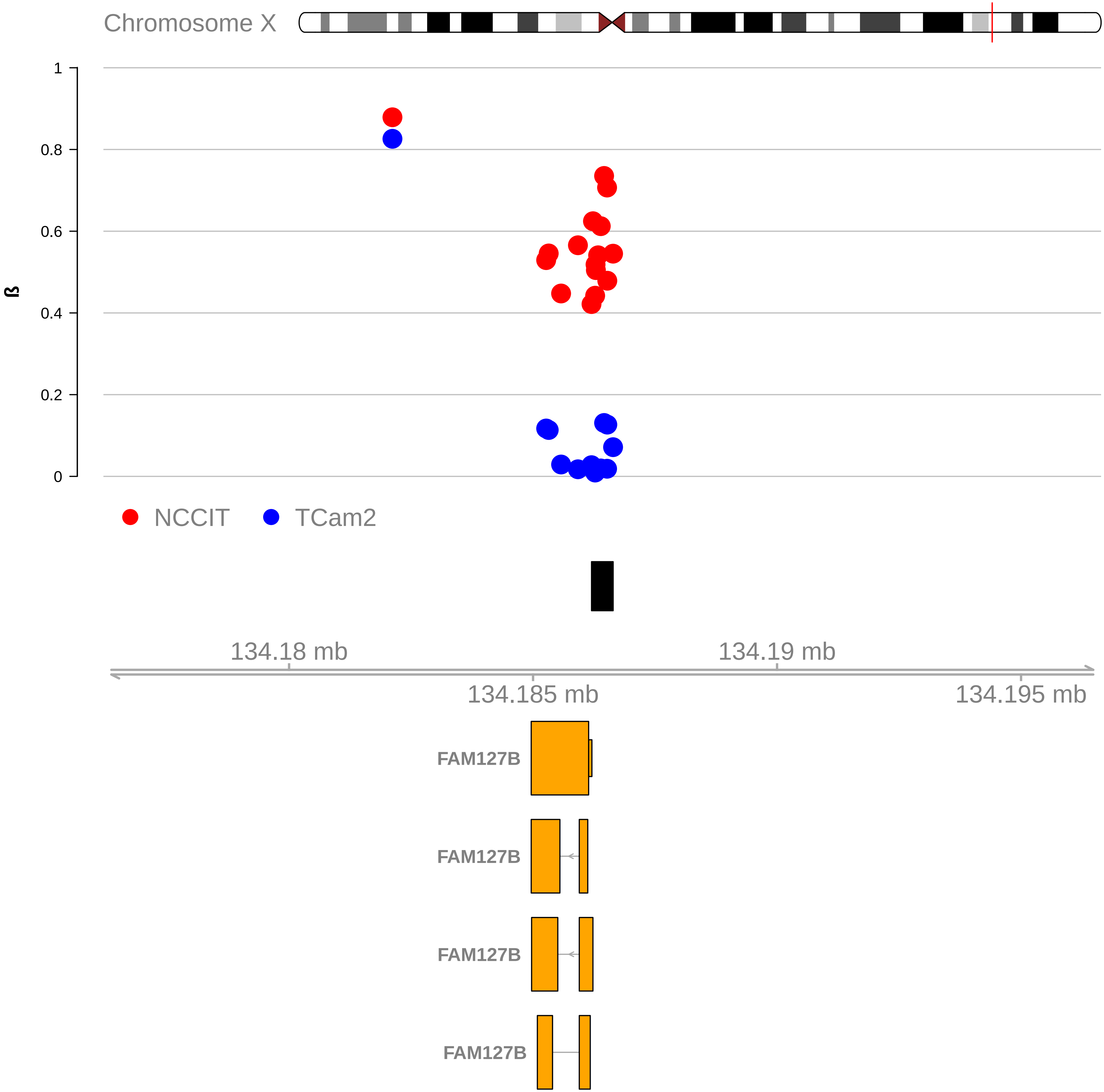

Chromosome X
1
0.8
0.6
0.4
0.2
0
ß
NCCIT
TCam2
134.18 mb
134.19 mb
134.185 mb
134.195 mb
FAM127B
FAM127B
FAM127B
FAM127B
